# Training and External Validation of a Predict Nomogram for Type 2 Diabetic Peripheral Neuropathy

**DOI:** 10.3390/diagnostics13071265

**Published:** 2023-03-27

**Authors:** Yongsheng Li, Yongnan Li, Ning Deng, Haonan Shi, Siqingaowa Caika, Gan Sen

**Affiliations:** 1 Department of Preventive Medicine, Medical College, Tarim University, Alar 843300, China; 2 Nursing Department, Suzhou BenQ Hospital, Suzhou 215163, China; 3 College of Biomedical Engineering and Instrument Science, Ministry of Education Key Laboratory of Biomedical Engineering, Zhejiang University, Hangzhou 310027, China; 4 College of Public Health, Xinjiang Medical University, Urumqi 830011, China; 5 The First Affiliated Hospital of Xinjiang Medical University, Urumqi 830054, China; 6 Department of Medical Engineering and Technology, Xinjiang Medical University, Urumqi 830011, China

**Keywords:** diabetic peripheral neuropathy, prediction model, nomogram, 25(OH)D3, type 2 diabetes mellitus (T2DM)

## Abstract

**Background:** Diabetic peripheral neuropathy (DPN) is a critical clinical disease with high disability and mortality rates. Early identification and treatment of DPN is critical. Our aim was to train and externally validate a prediction nomogram for early prediction of DPN. **Methods:** 3012 patients with T2DM were retrospectively studied. These patients were hospitalized between 1 January 2017 and 31 December 2020 in the First Affiliated Hospital of Xinjiang Medical University in Xinjiang, China. A total of 901 patients with T2DM from the Suzhou BenQ Hospital in Jiangsu, China who were hospitalized between 1 January 2019 and 31 December 2020 were considered for external validation. The least absolute shrinkage and selection operator (LASSO) and multivariate logistic regression were performed to identify independent predictors and establish a nomogram to predict the occurrence of DPN. The performance of the nomogram was evaluated using a receiver operating characteristic curve (ROC), a calibration curve, and a decision curve analysis (DCA). **Findings:** Age, 25-hydroxyvitamin D3 [25(OH)D3], Duration of T2DM, high-density lipoprotein (HDL), hemoglobin A1c (HbA1c), and fasting blood glucose (FBG) were used to establish a nomogram model for predicting the risk of DPN. In the training and validation cohorts, the areas under the curve of the nomogram constructed from the above six factors were 0.8256 (95% CI: 0.8104–0.8408) and 0.8608 (95% CI: 0.8376–0.8840), respectively. The nomogram demonstrated excellent performance in the calibration curve and DCA. **Interpretation:** This study has developed and externally validated a nomogram model which exhibits good predictive ability in assessing DPN risk among the type 2 diabetes population. It provided clinicians with an accurate and effective tool for the early prediction and timely management of DPN.

## 1. Introduction

Among chronic metabolic diseases with a high incidence and mortality, diabetes is one of the most widely recognized public health issues nowadays [1]. Statistics from the International Diabetes Federation reveal that the world developed 463 million new diabetes cases in 2019, and this figure is expected to rise to 700.2 million by 2045, with diabetes-related costs accounting for 12% of global healthcare consumption [2]. As the incidence of diabetes increases, the incidence of complications increases accordingly [3], with diabetic peripheral neuropathy (DPN) being one of the most common complications. As a neuropathy caused by chronic hyperglycemia [4,5], DPN is widely regarded as a major hazardous factor for foot ulcers and even lower limb amputations, and has an extremely high rate of disability and mortality, and a risk of death which even exceeds that of some cancers, for example, breast carcinoma and prostate adenocarcinoma [6]. Furthermore, the pathogenesis of DPN is exceptionally intricate, including multiple pathophysiological processes such as chronic inflammatory response [7], neurotrophic disorders [8], and oxidative stress [9], which cause DPN under the combined action of multiple variables. Unfortunately, there is no effective treatment for DPN in the medical community up to date. However, clinical symptoms in the early stages of DPN lack specificity, and the majority of patients have irreversible pathological changes in the peripheral nerves once symptoms such as limb numbness and pain occur [10]. Therefore, early diagnosis of this symptom remains a challenge in clinical practice.

Vitamin D is a fat-soluble hormone. Vitamin D deficiency is closely related to diabetic microangiopathy, and studies [11] have highlighted that vitamin D deficiency might be a critical cause of DNP. Moreover, numerous findings have shown that vitamin D deficiency has a significant impact on patients with DPN. Adverse conditions will increase the prevalence of DPN, leading to foot ulcers, lower limb amputations, and even death [6,11,12]. Since DPN is an irreversible disease, it is imperative to predict the likelihood of DPN, using serum 25 hydroxyvitamin D3 [25(OH)D3] (vitamin D in vivo in the form of 25(OH)D3) and associated risk factors, until type 2 diabetes mellitus (T2DM) has been identified as DPN. However, there have been few reports of correlation between the 25(OH)D3 factor and DPN, and there has never been an unprecedented risk prediction model based on 25(OH)D3 to predict the occurrence of DPN.

As a straightforward statistical visualization tool, nomograms have been broadly utilized in recent years to foresee the generation, development, prognosis, and endurance of diseases [13,14]. This study aims to establish a nomogram prediction model. The model incorporates a series of hazard factors for early prediction while aiding high-risk populaces to initiate timely interventions to diminish morbidity and mortality from diabetic peripheral neuropathy.

## 2. Methods

### 2.1. Study Design and Population

This is a retrospective analysis. The study protocol was approved by the Ethics Committees of the First Affiliated Hospital of Xinjiang Medical University (K202105-05) and Suzhou BenQ Hospital (SZMJYY2022102001) with a waiver for informed consent. The research was carried out in accordance with relevant guidelines and regulations.

In the training cohort, the retrospective study collected data from 3012 patients pathologically diagnosed with T2DM who were admitted to the First Affiliated Hospital of Xinjiang Medical University in Xinjiang, China, between 1 January 2017 and 31 December 2020. In the validation cohort were 901 patients with T2DM who were hospitalized in Suzhou BenQ Hospital in Jiangsu, China, from 1 January 2019 to 31 December 2020.

The inclusion criteria were as follows: (a) patients at least 18 years old, (b) the diagnostic criteria for T2DM was based on the Guideline for the prevention and treatment of type 2 diabetes mellitus in China (2013 edition) [15], and (c) all research participants were able to communicate independently. Exclusion criteria were as follows: (a) incomplete clinical data, and (b) peripheral neuropathy other than diabetic origin.

### 2.2. Data Collection

All baseline clinical characteristics were collected, including gender, age, white blood cell, total cholesterol, high-density lipoprotein, low-density lipoprotein, direct bilirubin, total bilirubin, aspartate aminotransferase, alanine aminotransferase, body mass index, systolic blood pressure, diastolic blood pressure, serum creatinine, hemoglobin A1c, glycosylated serum protein, apolipoprotein A1, apolipoprotein B, blood glucose, fasting blood glucose, 2-h postprandial blood glucose, triglyceride, blood urea nitrogen, urinary albumin/creatinine ratio, 25-hydroxyvitamin D3, cystatin C and homocysteine, diabetic peripheral neuropathy. (The diagnostic criteria for diabetic peripheral neuropathy was based on the Guideline for the prevention and treatment of type 2 diabetes mellitus in China (2013 edition)).

DPN: (a) abnormal temperature perception, (b) abnormal vibration perception, (c) nylon filament examination, foot sensation decreased or disappeared, (d) ankle reflex disappeared, and (e) nerve conduction velocity (NCV). If someone has two or more of the above phenomena, he/she is diagnosed as having DPN.

### 2.3. Training and Assessment of the Nomogram

First of all, for the training group, the optimal subset of predictors is obtained from the raw data utilizing the least absolute shrinkage and selection operator (LASSO) regression model [16], and multiple logistic regression analysis is used to select variables using LASSO regression to produce a 95% confidence interval (CI) odds ratio (OR) and associated *p*-values. Subsequently, a nomogram model was constructed to predict the risk of DPN based on the independent risk factors screened out by LASSO regression combined with multiple logistic regression. Following that, the prediction model was externally validated. In the end, the discrimination of the model was determined using the subject characteristic curve (ROC) and the area under the ROC curve (AUC) [17]. The Hosmer–Lemeshow goodness of fit test was used to assess the model fit and efficacy of the risk model [18]. Decision-curve analysis (DCA) was performed to determine the potential clinical effectiveness and benefits of the prediction model [19]. Internal validation was performed using the bootstrap resampling technique (1000 bootstrap samples).

### 2.4. Statistical Analysis

The continuous variables with normal distribution and homogeneity of variance are expressed as means ± standard deviations and tested using an independent sample *t*-test. For categorical variables, Chi-square analysis or Fisher’s exact test were used to compare the results. LASSO regression and multiple logistic regression were utilized to screen significant predictor variables. All statistical tests were two-tailed with a statistical significance level set at 0.05. R software (Version R-4.2.1; https://www.r-project.org accessed on 31 October 2022) were used for all statistical analyses.

## 3. Results

### 3.1. Patient Characteristics

The detailed baseline demographics and clinical characteristics of the patients in both cohorts are listed in Table 1. The baseline features were similar in both cohorts. For the training cohort, the clinical information for a total of 3012 patients was obtained from the First Affiliated Hospital of Xinjiang Medical University. The patients (1867 males and 1145 females) had a mean (SD) age of 57.12 ± 12.23 years of which the prevalence of DPN was 51.5%. For the external validation cohort, the clinical information for a total of 901 patients was obtained from Suzhou BenQ Hospital. The patients (573 males and 328 females) had a mean (SD) age of 56.60 ± 12.03 years, of which the prevalence of DPN was 45.7%.

### 3.2. Screening for Predictive Factors

**Figure 1:**
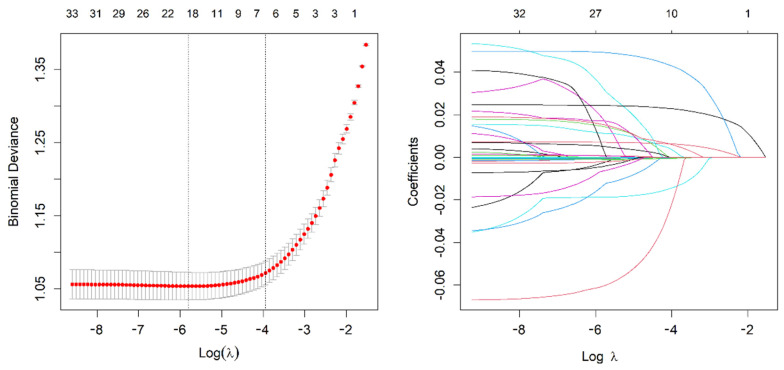
Demographic and clinical feature selection using the LASSO regression model.

We use the 3012 patients in the training cohort to choose features with nonzero coefficients in the LASSO regression model. The variables in the model were gradually lowered when the penalty coefficient was changed. Eventually, the 10-fold cross-validation error was chosen as the minimum λ + 1 (lambda. 1se = 0.019) and optimal value of the model; at this point, we have 6 variables (Figure 1). These potential predictors consisted of Age, 25(OH)D3, Duration of T2DM, HDL, HbA1c, and FBG. These six candidate variables were then analyzed using multivariate logistic regression. Ultimately, the six variables of Age (OR = 1.043, 95% CI (1.035, 1.052), *p* < 0.001); 25(OH)D3 (OR = 0.887, 95% CI (0.854, 0.921), *p* < 0.001); Duration of T2DM (OR = 1.151, 95% CI (1.134, 1.169), *p* < 0.001); HDL (OR = 0.993, 95% CI (0.99, 0.996), *p* < 0.001); HbA1c (OR = 1.309, 95% CI (1.251, 1.372); and FBG (OR = 1.060, 95% CI (1.027, 1.094), 0.003) were statistically significant and hence selected for the development of the prediction model (Table 2).

### 3.3. Risk Prediction Nomogram Development

**Figure 2:**
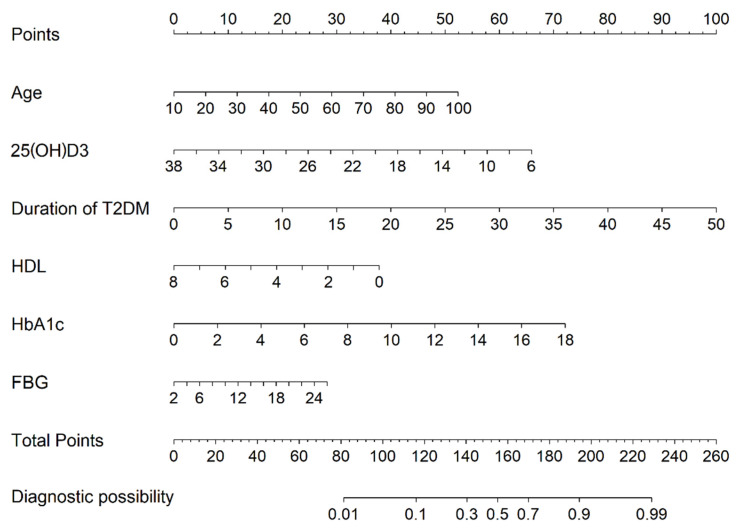
Nomogram for the perinatal prediction of DPN. DPN = diabetic peripheral neuropathy.

Based on the LASSO regression and multivariate logistic regression analysis and the clinical correlation results, seven predictors from the training cohort were included in the DPN risk prediction model and presented as a nomogram (Figure 2). The nomogram illustrated that each predictor corresponded to a specific score ranging from 0 to 100. The total score is determined by adding the scores of each predictor and is located on the “Total Points” axis. The probability of DPN corresponds to the bottom “Diagnostic possibility” axis of each patient. A higher score for a related factor in the nomogram indicates a higher risk of developing DPN.

### 3.4. Predictive Accuracy and Net Benefit of the Nomogram

**Figure 3:**
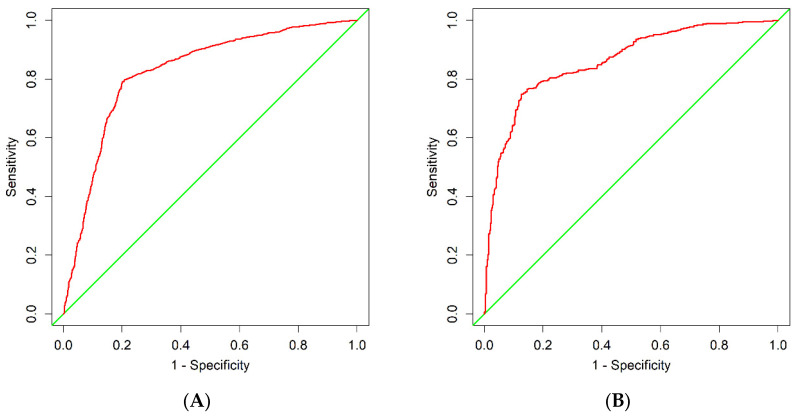
ROC curves. (**A**) Training cohort. (**B**) Validation cohort. ROC = receiver operating characteristic; AUC = area under the ROC curve.

**Figure 4:**
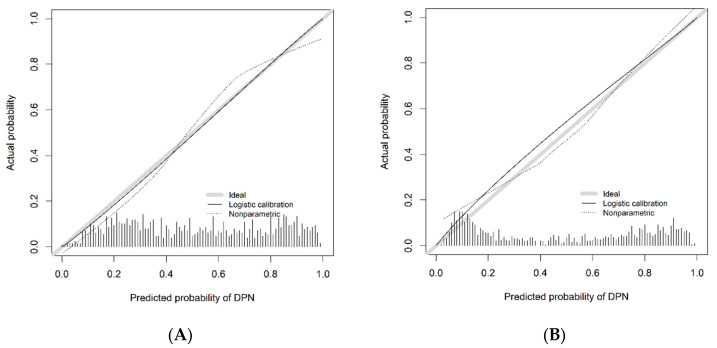
Calibration curve for predicting probability of DPN. (**A**) Training cohort. (**B**) Validation cohort. DPN = diabetic peripheral neuropathy.

For the training cohort, the area under the curve (AUC) was 0.8256 (95% CI: 0.8104–0.8408) (Figure 3A), and the calibration curve was close to the ideal diagonal line (Figure 4A). The Hosmer–Lemeshow test showed that the model was in line with the observed data (*p* > 0.05). In addition, 901 patients from Suzhou BenQ Hospital were used for external validation to test the nomogram. The AUC was 0.8608 (95% CI: 0.8376–0.8840) (Figure 3B), reflecting that the nomogram was accurate. Meanwhile, the model showed good consistency, and the calibration curve of the validation cohort was also close to the ideal diagonal line (Figure 4B). In addition, the Hosmer–Lemeshow test showed the model was in line with observed data (*p* > 0.05).

**Figure 5:**
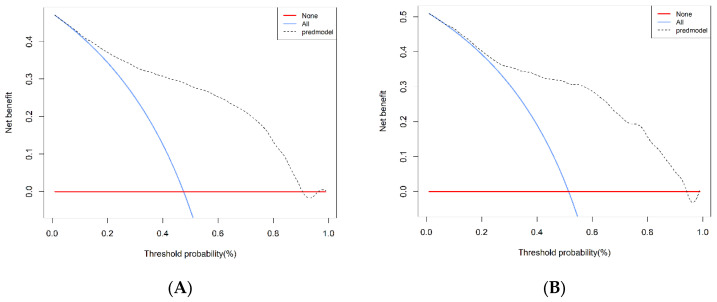
Decision curve analysis in prediction of DPN. (**A**) Training cohort. (**B**) Validation cohort. DPN = diabetic peripheral neuropathy.

The result of decision curve analysis for the nomogram is presented in Figure 5 (Net benefits for different threshold probabilities were shown in Table S1, Supplementary Material). In the training and validation cohorts, the decision curve showed that if the threshold probability of a patient was in the range of 0–0.90 and 0–0.94, using the model achieved more net benefits than the “full treatment” or “no treatment” strategy. There was a broad spectrum of alternative threshold probability, suggesting that the model was a good assessment tool.

## 4. Discussion

Diabetic peripheral neuropathy is one of the most common complications of diabetes. The International Diabetes League [2] survey revealed that the incidence of diabetic peripheral neuropathy is essentially as high as 30% to 50%. Additionally, once a diabetic patient develops neuropathy, the 5–10-year mortality rate is as high as 25% to 50%. Previous studies have reported the related factors of PDN, such as course of disease: hyperglycemia and hyperlipidemia, but a complete and comprehensive risk model for DPN assessment is still lacking. Our study is the first to include 25(OH)D3 in a risk prediction model to assess the risk of developing DPN in type 2 diabetes mellitus in China. The prediction model included six clinically available parameters, and the results showed that Age, 25(OH)D3, Duration of T2DM, HDL, HbA1c, and FBG were risk factors for DPN in T2DM. Additionally, the model will assist clinicians to identify high-risk individuals at an early stage and to apply appropriate interventions to improve the prognosis for T2DM.

In recent years, the relationship between vitamin D and microvascular complications of type 2 diabetes has attracted increasing attention from scholars at home and abroad. DPN is one of the main microvascular complications of diabetes. However, there is relatively little existing research available on the association of vitamin D with DPN. A retrospective study [20] comparing vitamin D deficiency and DPN in T2DM included 87 patients with DPN and 123 patients without DPN. The results found that the serum 25 (OH) D3 concentration in the DPN group was significantly lower than that in the DPN-free group. A total of 81% of patients in the DPN group suffered from vitamin D deficiency while 60.4% of the non-DPN group had a vitamin D deficiency. A similar cross-sectional study [11] discovered that the prevalence of DPN was comparable in the vitamin D-deficient and vitamin D-sufficient groups (31.89% vs. 31.80%). However, the prevalence of DPN increased to 46.63% in the vitamin D-deficient group. A case-control study conducted by Halawa M R et al. [21] in 178 prediabetic patients in Egypt demonstrated that vitamin D levels were inversely correlated with peripheral neuropathy severity (r = −0.47, *p* < 0.001). Unexpectedly, after vitamin D supplementation, the neuropathy score dropped from (Mean = 6.4, SD = 1.6) to (Mean = 2.5, SD = 0.9). In this study, vitamin deficiency and DPN correlation showed a strong significance, which is consistent with existing research. The most likely reason for this is that vitamins can promote the secretion of pancreatic B cells, reduce insulin resistance by increasing insulin secretion, improve blood sugar in patients, and protect the central nervous system to a certain extent. Dou X et al. [22] confirmed through animal experiments that 25(OH)D3 has a protective effect on the nerves of diabetic rats, while another animal experiment [23] demonstrated that the level of nerve growth factor in rats after the treatment of diabetic neuropathy with vitamin D3 derivatives was increased, suggesting that vitamin D can act on various cells of the nervous system and exert neuroprotective effects by regulating calcium homeostasis in neuronal cells. Meanwhile, a strong correlation between pro-inflammatory factors and DPN was found in the results of a 5-year prospective study in China [24], which was confirmed in a national health and nutrition examination survey study in the United States [25]. The researchers found that vitamin D can downregulate the expression of inflammatory factors such as tumor necrosis factor α (TNF-α), interleukin-6 (IL-6), and interleukin-1 receptor antagonist (IL-1RA), and can also inhibit the occurrence and development of inflammatory response, and thus can inhibit the occurrence of DPN. This imposes a requirement that in the future health management of DPN patients, attention should be paid to the monitoring of serum 25(OH)D3 and appropriate supplementation of vitamin D should be given. This requirement is expected to be one of the therapeutic means of preventing DPN and even accelerate the recovery of patients.

In most epidemiological studies of DPN, age and the course of diabetes are the most frequently evaluated immutable hazardous factors [3,26,27]. In a study of 60 hospitalized T2DM [28], the incidence of DPN in patients aged 20 to 34 years, 35 to 49 years, 50 to 64 years, and ≥ 65 years was found to be 8.4%, 22.7%, 33.0%, and 42.4%, respectively, and the incidence of DPN was significantly different from that of age. Studies by Popesco et al. [29] have shown a DPN prevalence of 28.8% based on the Michigan Neuropathy Screening Scale (MNSI) score, which is significantly positively associated with higher age. On the other hand, Skalli et al. [30] conducted a cross-sectional study of 111 T2DM, and subgroup analysis discovered that in patients with DPN, the older they were, the lower the 25(OH)D3 level. This conclusion was confirmed in a cross-sectional study of older patient populations in Shanghai [31]. The results of the study showed that vitamin D deficiency is prevalent in elderly T2DM, which also proves the presence of age-affected DPN. Chia-Tung et al. [32] and Zhang et al. [33] found that the longer the course of diabetes, the higher the prevalence of DPN, and it may be that neurofibropathy in patients with DPN leads to a decrease in fibrous nerve density, deepening the degree of skin denexylation and thus increasing the risk of DPN. Additionally, in a three-year follow-up study [34] in the Shihezi community in Xinjiang, it was mentioned that with the prolongation of the course of the disease, the function of pancreatic islet B cells decreased progressively. The UKPDS data from large studies shows that at the time patients are diagnosed with diabetes, nearly half of the function of secreting insulin has been lost, and the function of islet B cells in relation to the vitamin D diminished progressively [35]. However, the longer the course of the patient’s disease, the higher the age. Therefore, in clinical work, it is necessary to increase the screening of T2DM patients with T2DM with a longer course of disease and a higher age in order to slow or reduce the occurrence of DPN, thereby improving the quality of life of patients.

In existing studies, blood lipids, blood glucose, and the occurrence of DPN are also firmly related. Patients with T2DM often also suffer from disorders of the lipid metabolism, which are manifested by decreased HDL levels and elevated LDL levels. Studies by Yang et al. [36] have found that low HDL levels are a risk factor for the development of DPN in patients with T2DM. The reason for this may be that HDL promotes the reverse transport of cholesterol, accelerating TG and cholesterol metabolism, thereby stabilizing blood glucose concentrations. In one case-control study [37], HDL was positively correlated with nerve conduction velocity, and HDL levels were remarkably reduced in patients with DPN, suggesting that HDL may be a risk factor for DPN. The results of this study also suggest that the protective HDL is negatively correlated with DPN. Previous studies have speculated that the duration of diabetes can reflect the control of blood glucose, and HbA1c is a product of glucose-hemoglobin reaction, which can reflect the patient’s blood glucose level in the past 1 to 2 months. Mei Li [38] noted in the study that HbA1c levels in patients with DPN were significantly higher than in non-DPN patients, and that HbA1c was associated with the incidence of DPN. The greater the HbA1c level, the more common DPN is [39]. In this study, HbA1c levels were exceptionally positively correlated with DPN. Elevated HbA1c is an independent risk factor for the development of DPN, possibly because long-term hyperglycemia promotes non-enzymatic glycosylation of proteins on the nerve myelin sheath, leading to neurofibrillary signaling dysfunction [40], while elevated HbA1c levels indicate hyperglycemic status in patients with T2DM [41]. In that case, for patients with T2DM, it is necessary to control their blood glucose strictly and reduce the level of HbA1c, as this can effectively prevent and delay the occurrence of DPN. However, HbA1c levels may be affected by anemia, hemoglobin longevity, age, pregnancy, ethnicity, and other factors. It does not reflect rapid changes in blood sugar in the near term because it has a “delayed effect”. Hence some scholars believe that its reference value is limited. Despite this, microvascular patients with diabetes are more sensitive to blood glucose changes over a short period of time [42], and fasting blood glucose is the best test value. Our study is the first to discover that FBG is an independent risk factor for DPN. Although we note that for every 1 unit increase in FBG, OR is only 1.06, so the ability to predict the incidence of DPN is limited. Previous studies have not found FBG to be an independent risk factor for DR, something which may be related to the higher predictive value of HbA1c in these studies. Therefore, the possible mechanism of FBG treatment of DPN still needs to be further studied.

A nomogram is a statistical model of individualized predictive analysis of clinical events, and compared with other predictive statistical methods Nomogram analysis can provide better individualized predictive risk assessment in an intuitive and visual manner [13]. Our study identified the six risk factors screened out by LASSO regression combined with multivariate logistic regression analysis as routine clinical variables readily available to clinicians, making them easy to adopt in practice. Meanwhile, in this study, the overall prevalence of DPN was 50.1%, and the prevalence of DPN in Xinjiang was 51.5%, higher than the 45.7% in Suzhou, Jiangsu Province, which may be due to regional differences as the diet in Xinjiang is known to include more chili oil and salty in comparison to other regions. According to the results of the latest national cross-sectional study [43], Xinjiang has the highest prevalence of diabetes in in the country, which may be the reason for the high prevalence of DPN. By verifying the AUC area of the dataset (the AUC of the validation group is greater than the AUC of the training group), the model has a wide range of versatility. Furthermore, our external validation team confirmed the consistency of the model and its high net benefit, thus indicating that the prediction model is a good assessment tool that can provide some reasonable evidence in treating T2DM in China and can be used as a reference in the further management of DPN. Nevertheless, there are certain limitations to our research. Due to this study being a retrospective analysis, it was inevitable that there was a certain degree of internal bias, and whatsmore, samples from the training and validation cohort only represented the inland areas of China, and lacked samples from coastal areas. Consequently, we will seek validation evaluation in a multicenter study.

To summarize, we were the first to develop and externally validate a Nomogram that predicts the risk of developing DPN in T2DM using 25(OH)D3 as the major component. The external validation confirmed that the model is extremely accurate and displays favorable consistency, which can assist in early clinical intervention, and may be of substantial significance in diminishing the prevalence of and mortality from DPN in the future.

## Figures and Tables

**Table 1:** Baseline characteristics of all patients in the training cohort and validation cohort.

Variables	Training Cohort (n = 3012) Mean (SD)/N (%)	Validation Cohort (n = 901) Mean (SD)/N (%)	*p*
DPN (%)			0.003
No	1462 (48.5%)	489 (54.3%)	
Yes	1550 (51.5%)	412 (45.7%)	
Gender			0.381
Male	1867 (62.0%)	573 (63.6%)	
Female	1145 (38.0%)	328 (36.4%)	
Age (years)	57.123 (12.234)	56.600 (12.032)	0.259
WBC (×10^9^)	7.085 (2.395)	7.183 (2.845)	0.305
Neutrophil (×10^9^)	4.212 (2.024)	4.301 (2.206)	0.257
Eosinophil (×10^9^)	0.171 (0.161)	0.163 (0.133)	0.172
Lymphocyte (×10^9^)	2.168 (0.762)	2.160 (0.802)	0.788
Hemoglobin (g/L)	138.426 (18.945)	138.410 (19.365)	0.981
Platelet (×10^9^)	228.204 (67.877)	223.174 (66.781)	0.050
TC (mmol/L)	4.244 (1.149)	4.203 (1.057)	0.331
HDL (mmol/L)	1.108 (0.359)	1.089 (0.347)	0.160
LDL (mmol/L)	2.704 (0.889)	2.717 (0.874)	0.686
DB (U/L)	3.594 (2.051)	3.664 (2.309)	0.389
TB (U/L)	11.477 (5.730)	11.839 (6.139)	0.102
AST (U/L)	21.035 (14.277)	21.801 (16.145)	0.171
ALT (U/L)	25.506 (21.686)	26.677 (22.417)	0.158
BMI (kg/m^2^)	26.145 (3.797)	26.018 (3.780)	0.379
SBP (mmHg)	127.893 (16.966)	126.882 (16.226)	0.113
DBP (mmHg)	77.181 (10.099)	77.068 (9.875)	0.767
Duration of T2DM	8.351 (7.213)	8.255 (7.406)	0.728
Scr (μmol/L)	74.457 (30.558)	72.665 (32.855)	0.129
HbA1c (%)	8.748 (2.125)	8.643 (2.183)	0.196
GSP (%)	2.772 (0.716)	2.743 (0.698)	0.283
ApoA1 (g/L)	1.170 (0.253)	1.158 (0.249)	0.210
ApoB (g/L)	0.932 (0.288)	0.926 (0.279)	0.621
BG (mmol/L)	9.521 (4.844)	9.872 (5.485)	0.064
FBG (mmol/L)	8.867 (3.023)	8.762 (2.849)	0.354
PBG (mmol/L)	18.012 (4.528)	18.048 (4.533)	0.835
TG (mmol/L)	2.310 (2.270)	2.333 (2.020)	0.788
BUN (mmol/L)	6.981 (16.624)	6.198 (8.054)	0.172
UACR (mg/g)	38.52 ± 33.109	41.803 ± 37.233	0.011
25 (OH)D3 (ng/mL)	16.491 (8.908)	15.908 (6.823)	0.073
Cys C (mg/L)	2.332 (0.839)	2.227 (0.798)	0.081
Hcy (μmol/L)	12.713 (2.841)	12.871 (2.632)	0.137

Abbreviations: DPN: Diabetic peripheral neuropathy; M: Male; F: Female; WBC, white blood cell; TC: Total cholesterol; HDL: High-density lipoprotein; LDL: Low-density lipoprotein; DB: direct bilirubin; TB: total bilirubin; AST, aspartate aminotransferase; ALT, alanine aminotransferase; BMI: Body mass index; SBP: Systolic blood pressure; DBP: Diastolic blood pressure; Scr: Serum creatinine; HbA1c: hemoglobin A1c; GSP: glycosylated serum protein; ApoA1: apolipoprotein A1; ApoB: apolipoprotein B; BG: blood glucose; FBG: fasting blood glucose; PBG: 2-h postprandial blood glucose; TG: Triglyceride; BUN: Blood urea nitrogen; UACR: urinary albumin/creatinine ratio; 25(OH)D3: 25-hydroxyvitamin D3; Cys C: Cystatin C; Hcy: homocysteine.

**Table 2:** Multivariate Logistic Regression Analysis for Risk Factors of DPN.

Variables	OR	95% CI	*p*
Age	1.243	(1.235, 1.252)	<0.001
25(OH)D3	0.807	(0.754, 0.851)	<0.001
Duration of T2DM	1.351	(1.304, 1.379)	<0.001
HDL	0.903	(0.854, 0.957)	<0.001
HbA1c	1.309	(1.251, 1.372)	<0.001
FBG	1.06	(1.027, 1.094)	0.003

Abbreviations: 25(OH)D3: 25-hydroxyvitamin D3; HDL: high-density lipoprotein; HbA1c: hemoglobin A1c; FBG: fasting blood glucose.

## Data Availability

The data underlying this article will be shared on reasonable request to the corresponding author.

## Supplementary Materials

The following supporting information can be downloaded at: https://www.mdpi.com/article/10.3390/diagnostics13071265/s1; Table S1: Net benefits for different threshold probabilities.
