# Supplementary material for: Training and External Validation of a Predict Nomogram for Type 2 Diabetic Peripheral Neuropathy
Source: Diagnostics (Basel). 2023 Mar 27;13(7):1265. doi: 10.3390/diagnostics13071265 (PMC10093299; doi:10.3390/diagnostics13071265)
Supplement: Supplementary file 1 [file diagnostics-13-01265-s001.zip › diagnostics-2282715-supplementary.pdf]

diagnostics

Supplementary Material: Training and external validation of a predict nomogram for diabetic peripheral neuropathy among type 2 diabetes population

Yongsheng Li, Yongnan Li, Ning Deng, Haonan Shi, Siqingaowa·CaiKa, Gan Sen

Table S1. Net benefits for different threshold probabilities in the external validation cohort.

Threshold	All	None	Predmodel
**0.01**	0.50964951	0	0.50964951
**0.02**	0.504645933	0	0.504645933
**0.03**	0.49953919	0	0.49957134
**0.04**	0.494326057	0	0.494629245
**0.05**	0.489003173	0	0.488620199
**0.06**	0.483567037	0	0.483014111
**0.07**	0.478013994	0	0.479299399
**0.08**	0.472340233	0	0.474509627
**0.09**	0.466541774	0	0.470699778
**0.10**	0.460614461	0	0.468121968
**0.11**	0.454553949	0	0.460323764
**0.12**	0.448355698	0	0.454120204
**0.13**	0.442014959	0	0.446949602
**0.14**	0.435526761	0	0.442005512
**0.15**	0.428885899	0	0.438180262
**0.16**	0.422086922	0	0.429412929
**0.17**	0.415124114	0	0.421473837
**0.18**	0.407991481	0	0.416206075
**0.19**	0.400682734	0	0.408138908
**0.20**	0.393191268	0	0.402286902
**0.21**	0.385510145	0	0.395260402
**0.22**	0.37763207	0	0.39146543
**0.23**	0.36954937	0	0.383629884
**0.24**	0.361253967	0	0.378761352
**0.25**	0.352737353	0	0.373180873
**0.26**	0.34399056	0	0.368770017
**0.27**	0.33500413	0	0.362301712
**0.28**	0.325768076	0	0.360822361
**0.29**	0.316271851	0	0.359023162
**0.30**	0.306504307	0	0.356103356
**0.31**	0.296453644	0	0.353912441
**0.32**	0.286107374	0	0.353369206
**0.33**	0.275452261	0	0.34782946
**0.34**	0.264474264	0	0.345964846
**0.35**	0.253158484	0	0.343115305
**0.36**	0.241489085	0	0.341930873
**0.37**	0.229449229	0	0.342672343
**0.38**	0.217020991	0	0.340319227
**0.39**	0.20418527	0	0.336133738
**0.40**	0.190921691	0	0.332640333
**0.41**	0.177208499	0	0.330156101
**0.42**	0.163022439	0	0.327729586
**0.43**	0.148338622	0	0.324834956
**0.44**	0.133130383	0	0.320611821
**0.45**	0.117369117	0	0.322717823
**0.46**	0.101024101	0	0.321167321
**0.47**	0.084062292	0	0.318185384

**0.48**	0.066448105	0	0.32016632
**0.49**	0.048143166	0	0.31655864
**0.50**	0.029106029	0	0.313929314
**0.51**	0.009291866	0	0.311404811
**0.52**	-0.011347886	0	0.305959806
**0.53**	-0.032865926	0	0.303578538
**0.54**	-0.055319534	0	0.308370243
**0.55**	-0.078771079	0	0.306306306
**0.56**	-0.103288603	0	0.302967303
**0.57**	-0.128946478	0	0.298167577
**0.58**	-0.155826156	0	0.298881299
**0.59**	-0.184017038	0	0.291161706
**0.60**	-0.213617464	0	0.285862786
**0.61**	-0.24473586	0	0.284796631
**0.62**	-0.277492067	0	0.273662326
**0.63**	-0.31201888	0	0.268977918
**0.64**	-0.348463848	0	0.262531763
**0.65**	-0.386991387	0	0.257944758
**0.66**	-0.427785251	0	0.248318454
**0.67**	-0.471051471	0	0.23965224
**0.68**	-0.51702183	0	0.227780665
**0.69**	-0.565958018	0	0.22563879
**0.70**	-0.618156618	0	0.215523216
**0.71**	-0.673955122	0	0.213742921
**0.72**	-0.733739234	0	0.199138699
**0.73**	-0.797951798	0	0.196735197
**0.74**	-0.86710379	0	0.194786502
**0.75**	-0.941787942	0	0.193347193
**0.76**	-1.022695773	0	0.191961192
**0.77**	-1.110639067	0	0.191268191
**0.78**	-1.206577207	0	0.183991684
**0.79**	-1.311652312	0	0.168399168
**0.80**	-1.427234927	0	0.159043659
**0.81**	-1.554984134	0	0.140606193
**0.82**	-1.696927697	0	0.135250635
**0.83**	-1.855570503	0	0.130610248
**0.84**	-2.034043659	0	0.116943867
**0.85**	-2.236313236	0	0.114345114
**0.86**	-2.467478467	0	0.097119097
**0.87**	-2.73420758	0	0.085319047
**0.88**	-3.045391545	0	0.079348579
**0.89**	-3.413154413	0	0.071064071
**0.90**	-3.854469854	0	0.055093555
**0.91**	-4.393855394	0	0.040887041
**0.92**	-5.068087318	0	0.046257796
**0.93**	-5.934956935	0	0.026136026
**0.94**	-7.090783091	0	0.004158004
**0.95**	-8.708939709	0	-0.021829522
**0.96**	-11.13617464	0	-0.023908524
**0.97**	-15.18156618	0	-0.054747055
**0.98**	-23.27234927	0	0.003118503
**0.99**	-47.54469855	0	0.002079002
